# Video event data recording of a taxi driver used for diagnosis of epilepsy^☆^

**DOI:** 10.1016/j.ebcr.2013.12.007

**Published:** 2014-01-24

**Authors:** Kotaro Sakurai, Junko Yamamoto, Tsugiko Kurita, Youji Takeda, Ichiro Kusumi

**Affiliations:** Department of Psychiatry and Neurology, Hokkaido University School of Medicine, Sapporo, Japan

**Keywords:** Video event data recorder, Epilepsy, Driving

## Abstract

A video event data recorder (VEDR) in a motor vehicle records images before and after a traffic accident. This report describes a taxi driver whose seizures were recorded by VEDR, which was extremely useful for the diagnosis of epilepsy. The patient was a 63-year-old right-handed Japanese male taxi driver. He collided with a streetlight. Two years prior to this incident, he raced an engine for a long time while parked. The VEDR enabled confirmation that the accidents depended on an epileptic seizure and he was diagnosed with symptomatic localization-related epilepsy. The VEDR is useful not only for traffic accident evidence; it might also contribute to a driver's health care and road safety.

## 1 Introduction

A video event data recorder (VEDR) is a device that records images before and after a traffic accident. In Japan, many taxicabs have been equipped with a VEDR, which records not only the car exterior but also the interior for crime prevention. Video event data recorder records are increasingly used as evidence related to traffic accidents and crimes.

This report describes a taxi driver whose seizures were recorded by a VEDR. That information was useful for the diagnosis of epilepsy.

## 2 Case report

The patient was a 63-year-old right-handed Japanese man with normal psychomotor development. At age 56, unruptured cerebral aneurysms in the left anterior communicating artery, internal carotid-posterior communicating artery, and internal carotid-anterior choroidal artery were discovered. Neurosurgical clipping was done.

He reported no history of cardiovascular disease or diabetes mellitus. He consumed no alcohol. He was single and had worked as a taxi driver from age 50. At age 61, a strange episode occurred during which he continued racing the engine for a long time while parked. Furthermore, after a rear-end collision in another year, he reportedly did not reply to questions of other people for some time.

At age 63, he collided with a streetlight. Although he was uninjured, he was taken by ambulance to a nearby neurosurgical hospital because of unconsciousness. At the time of his arrival at the hospital, he had already become lucid. Brain MRI, MRA, an electrocardiogram, and a blood test showed no abnormality. He was discharged from the hospital one week later and was referred to our hospital by his work supervisor for suspicion of epilepsy. Although he remembered having stopped at the crossing, he had no subsequent memory of the event. He said that he did not understand what had caused the accident. However, he said that the accident was not based on illness but might be attributable to dozing. Regarding the episode of racing the engine, he said that he had stomach cramps and had stepped on the accelerator and brake at the same time.

Because the taxi company used VEDR, images at the time of the crash accident were available (Video 1), as were those at the time he raced the engine for a long time two years prior (Video 2).

In Video 1, he had stopped at a red light. However, he bowed his head after a while. The taxi began to move forward slowly in spite of the red light. Apparently, the brake was taken off because he lost consciousness. The taxi, with automatic transmission, crept forward. The taxi moved into the intersection and crashed into a streetlight. A pedestrian spoke to him soon after the crash, but he was unresponsive. Clear convulsions were not confirmed in this record.

In Video 2, he had parked while waiting for passengers. The onset of the seizure was not clear, but he took a slouching posture. Subsequently, racing of the engine began with convulsions of both arms. His neck and trunk turned slowly to the right side. The convulsions stopped once, but generalized clonic convulsions began after 38 s. His body began swinging back and forth intensely. This clear convulsion stopped 33 s later. However, he continued racing the engine intermittently for 3 min. A driver who had parked behind noticed the strange situation. When the driver called, “Is your car all right?”, he responded, “Yes”, but he continued racing the engine for 2 min. When he was directed to stop the engine, he did not reply but stopped the engine nonetheless. The record by the VEDR also stopped at that time because the engine had been turned off. After 37 min, he already regained consciousness. He was able to drive again and had no problem speaking with his supervisor on a mobile phone.

**Fig. 1:**
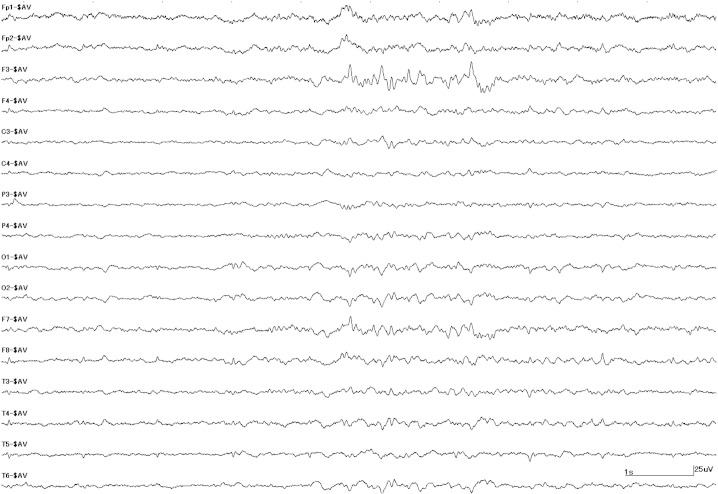
Interictal EEG. EEG spikes are visible in the left frontal area.

Thus, the VEDR record confirmed a loss of consciousness, convulsion, and a postictal confused state. Scalp interictal EEG at our institution showed repetitive left frontal spikes (Fig. 1). From this information, we ascertained that the accidents had been dependent on epileptic seizures and diagnosed him as having symptomatic localization-related epilepsy. We explained epilepsy to him and prohibited him from driving because being seizure-free for more than two years is necessary for driving in Japan.

## 3 Discussion

This report described a taxi driver whose epileptic seizures had been recorded by VEDR. Because VEDR records provided important information, we were able to diagnose his epilepsy. Moreover, we were able to take necessary measures of medical intervention and driving prohibition.

Motor vehicle crashes associated with medical conditions present important problems for road safety. Recently, Charlton et al. reviewed studies of medical conditions and crashes, which revealed that eight conditions have at least a moderately elevated risk (relative risk: 2.1–5.0) of crash involvement compared with their relevant control group. These were alcohol abuse, dementia, epilepsy, multiple sclerosis, psychiatric disorders (regarded as a group), schizophrenia, sleep apnea, and cataracts [1]. However, it was also found that well-established treatments can reduce risk to the same level as those without the condition [1]. Therefore, early detection of illness and medical intervention are extremely important.

As our case showed, VEDR records can provide useful information related to medical diagnosis and motor vehicle accidents. Video event data recorder records are available not only as traffic accident evidence; they might also contribute to a driver's health care and road safety.

The following are the supplementary data related to this article.

Video 1 The VEDR record of the crash. The upper half of the video shows footage taken by the exterior camera, and the bottom half of the video shows footage taken by the interior camera. News is broadcast on the radio in the car.

Video 2 The VEDR record of racing of the engine (interior camera). News is broadcast on the radio in the car.
