# Supplementary figures and images for: Video event data recording of a taxi driver used for diagnosis of epilepsy
Source: Epilepsy Behav Case Rep. 2014 Jan 24;2:24–5. doi: 10.1016/j.ebcr.2013.12.007 (PMC4307877; doi:10.1016/j.ebcr.2013.12.007)

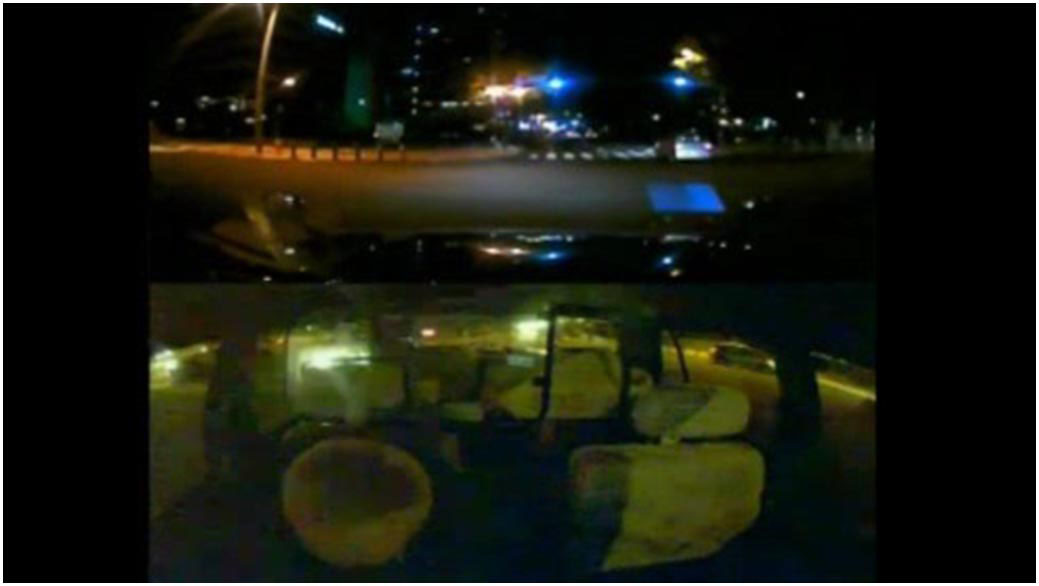

Supplement: Video 1 — The VEDR record of the crash. The upper half of the video shows footage taken by the exterior camera, and the bottom half of the video shows footage taken by the interior camera. News is broadcast on the radio in the car. [file mmc1.jpg]

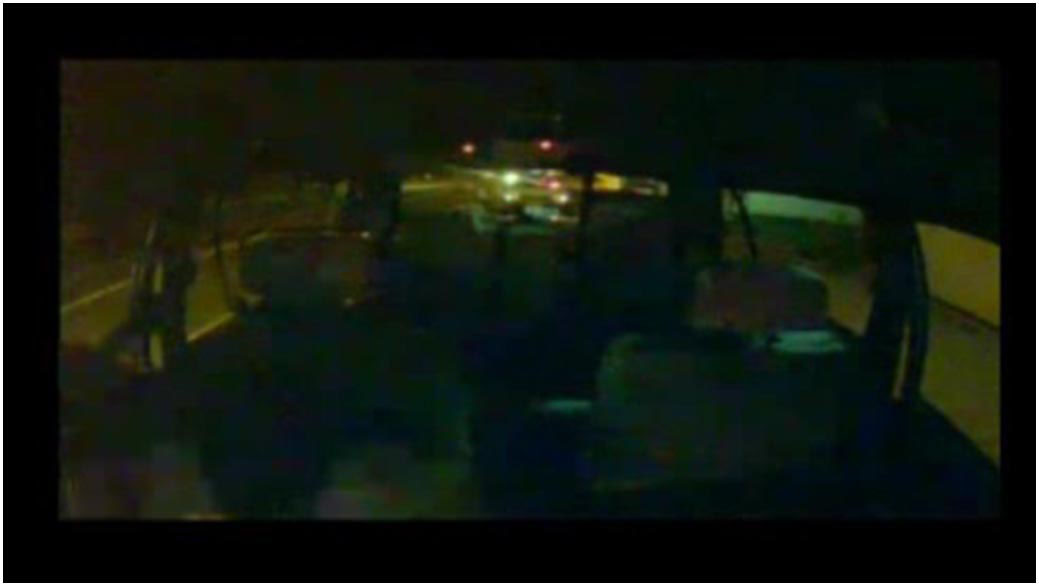

Supplement: Video 2 — The VEDR record of racing of the engine (interior camera). News is broadcast on the radio in the car. [file mmc2.jpg]
